# Operational Validity in Decentralized Molecular Point-of-Care Diagnostics: A Human Factors Engineering Perspective

**DOI:** 10.3390/diagnostics16121924

**Published:** 2026-06-21

**Authors:** Moustafa Kardjadj

**Affiliations:** dicentra, Toronto, ON M4W 3E2, Canada; drkardjadj@live.fr

**Keywords:** human factors engineering, molecular point-of-care, operational validity, usability engineering, decentralized testing

## Abstract

The rapid expansion of molecular point-of-care (POC) diagnostics into decentralized settings, including emergency departments, retail pharmacies, and home environments, has shifted the burden of diagnostic performance from laboratory professionals to heterogeneous, often non-expert users. While traditional evaluation frameworks focus on analytical and clinical validity, they often overlook the impact of human-system interactions on real-world reliability. This review introduces the concept of Operational Validity: the ability of a diagnostic system to preserve its intended performance when operated by intended users within the constraints of real-world workflows and environments. To establish a rigorous foundation for this concept, this study provides a critical comparative analysis contrasting Operational Validity against traditional clinical evaluation dimensions (analytical validity, clinical validity, and clinical utility) and post-market metrics. While existing literature outlines isolated usability principles, the significance of this study lies in its synthesis of these fragmented concepts into a formalized, lifecycle-based “Operational Validity” framework that explicitly maps the causal mechanisms connecting initial user interaction directly to downstream clinical outcomes. By synthesizing international standards (IEC 62366-1) alongside the newly finalized May 2026 U.S. Food and Drug Administration (FDA) guidance on the Content of Human Factors Information in Medical Device Marketing Submissions, we examine how human factors engineering (HFE) and usability engineering serve as the methodological foundation for operational validity. We analyze the specific complexities of molecular workflows, identify key parameters of use-related failure modes in pre-analytical and interpretation stages, and detail the mandatory role of iterative formative and final summative usability testing in mitigating these risks. Finally, we propose a lifecycle-based approach to HFE that integrates design, simulated-use validation, and post-market surveillance. Establishing operational validity is essential to ensure that the high analytical sensitivity of molecular POC platforms translates into consistent clinical utility across the full spectrum of decentralized care.

## 1. Introduction: POC Expansion, Traditional Validation, and the Emergence of Operational Validity

The evaluation of diagnostic tests has traditionally been organized around three linked dimensions: analytical validity, clinical validity, and clinical utility [1,2,3]. Analytical validity addresses whether an assay measures the intended target accurately in the test system; clinical validity addresses whether the result correctly identifies the clinical condition of interest; and clinical utility addresses whether test use improves decisions, outcomes, or resource use in practice [1,2,3]. This framework remains essential, but it does not fully capture what happens when a diagnostic is moved from a controlled laboratory into decentralized settings where heterogeneous users perform testing under variable conditions [3,4,5].

This limitation is especially critical for point-of-care (POC) molecular diagnostics. These systems are increasingly deployed in emergency departments, outpatient clinics, pharmacies, community sites, and home settings, where users may not be laboratory professionals and where the use environment is rarely standardized [4,5]. To systematically address these field vulnerabilities, we must explicitly define how Operational Validity differs from, and complements, traditional frameworks and related concepts:

- Analytical Validity vs. Operational Validity: Analytical validity establishes whether an assay can detect a target under optimized laboratory conditions [1,2,3]. Operational validity evaluates whether that internal capability is preserved when a non-expert operator executes the sampling and cartridge-loading protocols under real-world stressors [4,5].
- Clinical Validity vs. Operational Validity: Clinical validity links a test result to a specific clinical condition [1,2,3]. Operational validity ensures that user-induced errors (e.g., pre-analytical collection mistakes) do not degrade sample quality, which would otherwise alter the test’s field sensitivity or specificity [4,5].
- Clinical Utility vs. Operational Validity: Clinical utility determines if test use improves clinical decisions and patient outcomes [1,2,3]. Operational validity is the baseline prerequisite ensuring that the user interface does not cause delayed or misinterpreted results that sabotage those clinical decisions [4,5].
- Usability Validation and Human Factors Engineering (HFE): These represent the formal engineering methods, testing protocols, and design frameworks used during device development to systematically identify and eliminate use-related risks [6,7].
- Real-World Performance and Post-Market Surveillance: These represent the observational, longitudinal metrics and field data collected *after* commercial deployment to track whether operational validity is successfully maintained over time [2,3,8].

Human factors engineering and usability engineering were developed to address these risks precisely by aligning the device, the user interface, the intended users, and the intended use environment [6,7]. FDA guidance emphasizes safe and effective use in the intended users, uses, and environments, while the International Electrotechnical Commission (IEC 62366-1) formalizes a usability engineering process focused on normal use and use-related risk [6,7].

On this basis, this review proposes the concept of operational validity: the degree to which a diagnostic system preserves its intended performance under real-world use conditions, by intended users, in intended environments, and within realistic workflow constraints. Operational validity does not replace analytical validity, clinical validity, or clinical utility; rather, it bridges laboratory performance and field performance. For POC molecular diagnostics, operational validity depends on the integrity of specimen collection, sample transfer, cartridge loading, software navigation, timing, result interpretation, and reporting, all of which may be degraded by interruptions, time pressure, limited training, noise, or cramped workspaces [4,5,6,7].

The rationale for this review is straightforward: a POC molecular assay can be analytically excellent and even clinically relevant, yet still fail in practice if users cannot execute the workflow reliably or interpret the output correctly under real-world conditions [4,5,6,7]. While the existing literature outlines isolated usability principles [4,5,9], this work provides a new and highly significant contribution by prioritizing validation on steps like sampling and interpretation and synthesizing these concepts into a formalized “Operational Validity” framework. This article explicitly maps out the causal mechanism connecting initial user interaction directly to downstream clinical outcomes.

The objective of this review is to synthesize the current evidence on human factors engineering and usability validation in decentralized molecular diagnostics, to position operational validity as a useful conceptual layer between test performance and clinical impact, and to define how simulated-use and summative usability studies should be designed to reflect actual practice. The aim is to provide a framework that connects regulatory expectations, engineering design, and clinically meaningful diagnostic performance in decentralized care settings [4,5,6,7].

The urgency of establishing this framework is underscored by the current trajectory of the medical device industry. Modern point-of-care tools have rapidly transitioned from basic manual readouts into highly complex, automated platforms [2,5]. Recent breakthroughs have successfully integrated advanced biofluidics with computational intelligence, utilizing AI/ML-driven mechanisms to directly guide high-stakes clinical decisions [10]. Concurrently, the operational environment has diversified as decentralized molecular assays are deployed globally to monitor acute health threats, such as tracking neglected infectious diseases and surfacing emerging drug-resistance mechanisms at the point of care [11]. Moving these highly sophisticated biochemical testing steps into the field makes a lifecycle-based approach to operational validity paramount to ensuring diagnostic accuracy.

### Literature Search and Selection Strategy

Because this manuscript is framed as a perspective review analyzing the intersection of human factors engineering and real-world molecular diagnostic performance, a targeted literature search was conducted across major databases including PubMed, Embase, and Google Scholar for articles published through 2026. The search strategy utilized targeted boolean logic combining keywords such as: (“operational validity” OR “usability validation” OR “human factors engineering”) AND (“molecular point-of-care” OR “decentralized diagnostics” OR “near-patient testing”). Selection criteria prioritized peer-reviewed methodological frameworks and international consensus usability engineering standards, including IEC 62366-1 and related device validation architectures. Additionally, the selection process incorporated official regulatory guidance documents from the U.S. Food and Drug Administration (FDA) regarding premarket human factors engineering, over-the-counter testing parameters, and direct-to-consumer implementation. Notably, this review incorporates the final 2026 FDA guidance on the “Content of Human Factors Information in Medical Device Marketing Submissions,” which establishes the current regulatory requirements for documenting HF information in 510(k), De Novo, PMA, and HDE applications. Finally, the strategy integrated diagnostic evaluation models alongside recent peer-reviewed clinical evaluations and field studies. These selections were chosen specifically because they provide robust empirical documentation regarding user-induced pre-analytical sampling errors, device vulnerabilities to ambient environmental stressors, and workflow-driven turnaround time delays or operational deviations within decentralized, non-laboratory testing environments.

## 2. Human Factors Engineering Principles in Medical Devices

Human factors engineering (HFE) and usability engineering (UE) characterize the multifaceted interaction between the user, the device, and the use environment. These disciplines focus on how interface design dictates perception, cognitive processing, and physical execution during device setup, operation, and maintenance [6,8]. Within the medical-device framework, the primary objective of HFE is to systematically minimize use-related hazards and provide objective evidence that the device can be used safely and effectively by the intended user population within the intended settings [6,8]. This is particularly critical for decentralized diagnostics, where the operator profile shifts from specialized laboratory technologists to a heterogeneous group including nurses, pharmacists, community health workers, or patients, and where the environment may range from high-acuity emergency departments to the unstandardized conditions of the home [4,5,8].

From a methodological perspective, HFE transforms use-related risk into tangible design requirements that facilitate correct task completion while buffering against human error [6,8,9]. Fundamental principles such as cognitive ergonomics, interface simplicity, unambiguous feedback, and “error-proofing” (poka-yoke) are not merely aesthetic considerations; they are functional determinants of whether a user can reliably unpack, prime, and interpret a diagnostic system under the stressors of actual practice [6,8]. For molecular point-of-care (POC) diagnostics, the necessity of HFE remains high despite increasing automation. Even “sample-to-answer” platforms require precise specimen handling, complex cartridge loading, and nuanced software interaction, all of which are susceptible to failure if there is a mismatch between device complexity and user capability [4,5].

**Figure 1:**
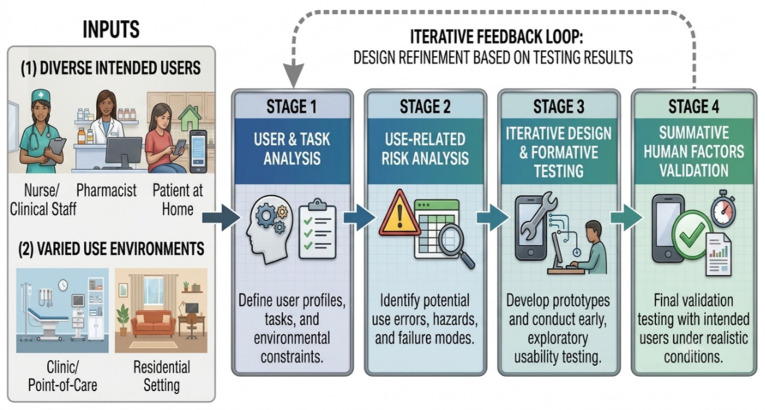
The Human Factors Engineering (HFE) Lifecycle for Decentralized Diagnostics. This figure illustrates the four-stage HFE process aligned with 2026 FDA submission requirements. Beginning with “INPUTS” (diverse users and environments), the process proceeds sequentially: Stage 1 (User & Task Analysis) defines user needs and environmental constraints; Stage 2 (Use-Related Risk Analysis) identifies human errors and safety consequences; Stage 3 (Iterative Design & Formative Testing) refines prototypes for usability; and Stage 4 (Summative Human Factors Validation) confirms the device meets all design requirements for regulatory approval.

The regulatory landscape, governed by the FDA’s finalized 2026 guidance “Content of Human Factors Information in Medical Device Marketing Submissions” and the international standard IEC 62366-1, frames HFE as a mandatory, rigorous lifecycle process. The FDA now explicitly mandates that HFE documentation in 510(k), De Novo, PMA, and HDE applications must demonstrate that use-related risks are identified, mitigated, and validated through robust summative testing. While IEC 62366-1 provides the structured process spanning from initial use-specification to summative validation, the current FDA expectations require a seamless integration between formative refinement and final safety proof. In practice, these frameworks mandate a systematic progression from iterative formative testing to final summative validation (Figure 1) [6,7,9].

In the context of decentralized molecular diagnostics, HFE serves as the methodological cornerstone of operational validity. While analytical validity confirms that the assay chemistry functions under idealized conditions, HFE validates that the diagnostic system can be executed with high fidelity by real-world users in chaotic environments. Consequently, usability engineering is not an adjunct to diagnostic performance; it is the mechanism by which real-world diagnostic integrity is maintained [4,5,6,7,8,9].

## 3. POC Molecular Diagnostic Platforms and Workflow Complexity

Point-of-care (POC) molecular diagnostics compress traditionally laboratory-bound workflows into compact, near-patient systems. However, rather than eliminating complexity, these platforms redistribute it across a sequence of user-dependent steps that must be executed with high fidelity in decentralized settings [4,5]. Across cartridge-based and microfluidic platforms, the core workflow typically encompasses specimen collection, sample preparation and transfer, cartridge loading, instrument initiation, and result interpretation [5,10,11].

This structure highlights a critical paradox: as automation increases within the “black box” of the instrument, the relative importance of the remaining manual steps increases. Even in highly automated “sample-to-answer” systems, the user remains the primary bridge between the biological specimen and the analytical core. Consequently, diagnostic performance is not merely a feature of assay chemistry; it is an emergent property of the integrated user–device–workflow system [4,5,10,11].

From a human factors perspective, this workflow introduces multiple vulnerable, use-related risk nodes. Each transition requires a combination of physical coordination (e.g., volumetric precision during pipetting, correct cartridge orientation), cognitive processing (e.g., adhering to timed steps, navigating hierarchical software menus), and environmental adaptation (e.g., maintaining sterility in a non-sterile field). Failures at any of these nodes can propagate through the system, often in ways that the instrument cannot detect. For instance, sub-optimal specimen collection may reduce the starting analyte concentration below the limit of detection, while incorrect software navigation may lead to the selection of an inappropriate test profile, both resulting in clinically misleading outcomes [10,11,12].

The complexity of molecular POC systems is further illuminated when contrasted with simpler formats such as lateral flow assays (LFAs). While LFAs are susceptible to interpretation errors, they generally impose a significantly lower cognitive and operational burden on the user [5,12]. Molecular platforms, by contrast, often require strict adherence to procedural sequencing and sophisticated interface interaction. As the analytical core becomes more “invisible” through automation, the upstream user-controlled steps, such as specimen lysis or transfer, become the most significant determinants of whether the test achieves its laboratory-validated potential [4,5,10,11].

Ultimately, operational validity in POC molecular diagnostics is contingent upon the reliability of this entire system. Identifying where these use-related risks reside is essential for defining the parameters of usability validation (Table 1).

## 4. Operational Validity Framework: Determinants and Impact on Diagnostic Performance

Human factors directly modulate diagnostic performance by dictating the fidelity with which a test is executed in the field. In molecular point-of-care (POC) systems, analytical validity is not an intrinsic, immutable property of the assay; rather, it is conditional upon the correct execution of a multi-step workflow. Consequently, use-related errors do not merely result in “user dissatisfaction”; they introduce stochastic variability that can degrade effective sensitivity, compromise specificity, elevate invalid-run rates, and generate clinically misleading results [1,2,3,4,5].

The pre-analytical phase serves as a primary locus of this functional dependency. Molecular assays are exquisitely sensitive to the integrity of the starting material; however, in decentralized settings, the “lab-grade” specimen is replaced by samples collected under variable conditions [10]. Empirical evidence from the SARS-CoV-2 pandemic demonstrated that suboptimal biological sampling was a significant driver of false-negative outcomes [11]. Furthermore, anatomical misunderstandings and technique-related variance during swabbing can substantially reduce the effective limit of detection (LoD) of an otherwise robust assay [12]. These data underscore that even a perfectly optimized molecular reagent will fail to deliver clinical value if the human-dependent workflow is compromised [10,11,12].

Beyond the specimen, environmental and operational stressors introduce additional layers of risk. Decentralized reagents and instrumentation often operate near the boundaries of their validated tolerances for temperature and humidity, which can impact both chemical stability and mechanical fluidics [13]. Field studies indicate that real-world diagnostic performance is frequently shaped by how seamlessly a device integrates into the chaotic clinical workflow, such as the presence of cognitive “noise” or physical space constraints, rather than by the specifications listed in the manufacturer’s instructions for use [14].

Synthesizing these elements, we define Operational Validity as follows:

*The ability of a diagnostic system to preserve its validated analytical and clinical performance when operated by intended users, in intended environments, under the constraints of real-world workflows.*

Operational validity acts as the functional bridge between the idealized laboratory environment and the decentralized field. It emerges from the dynamic interaction of four primary determinants: the User, the Device Interface, the Workflow, and the Environment. In this framework, performance degradation is rarely the result of a single “failure point” but rather a misalignment across the system. Therefore, while analytical excellence is a prerequisite, it is not a guarantee of diagnostic efficacy; reliable outcomes are ultimately contingent upon the system’s resilience to human and environmental variability (Table 2) [1,2,3,4,5,13,14].

## 5. Regulatory Expectations: FDA and IEC 62366-1

Regulatory frameworks for medical devices explicitly recognize usability as a core determinant of safety and effectiveness, particularly for point-of-care (POC) diagnostics deployed in decentralized settings. Both the U.S. Food and Drug Administration (FDA) and the International Electrotechnical Commission (IEC 62366-1) require manufacturers to implement a structured usability engineering process that integrates human factors into device design, risk management, and validation [6,7,8,9].

These frameworks reflect a fundamental evolution in diagnostic evaluation: performance must not only be demonstrated under controlled conditions but also under the stressors of intended use. Although not explicitly labeled as such, regulatory expectations implicitly target operational validity by requiring objective evidence that devices can be used reliably by intended users, in intended environments, and within realistic workflows (Table 3).

### 5.1. Usability Engineering as a Lifecycle Process

Both FDA guidance and IEC 62366-1 define usability engineering as a systematic, lifecycle-based process rather than a discrete event. This process begins with the characterization of intended users and use environments, followed by a detailed Task Analysis to understand how the device will be operated in practice [16,17,18].

A central requirement is the identification of critical tasks; user actions that, if performed incorrectly or omitted, could result in harm or compromised diagnostic performance [6,16]. In molecular POC diagnostics, these critical tasks are often “hidden” in the pre-analytical phase, including specimen homogenization, precise volumetric transfer, and the correct seating of the test cartridge. IEC 62366-1 formalizes this by requiring a Usability Engineering File that documents hazard-related use scenarios, ensuring that usability is treated as a primary design input rather than a downstream consideration [7,18].

### 5.2. Risk Analysis of Use Errors

A defining feature of modern regulation is the treatment of “human error” as a foreseeable and analyzable risk. FDA guidance requires manufacturers to identify use-related hazards and implement design controls to mitigate them, effectively shifting the burden of success from the user’s “carefulness” to the device’s “robustness” [6,16]. Similarly, IEC 62366-1 links usability directly to risk management (ISO 14971), requiring that residual risks be evaluated and reduced to acceptable levels [7,9,18]. For decentralized diagnostics, this means the system must be designed to be “forgiving” of the interruptions and environmental noise typical of pharmacy or home settings.

### 5.3. Formative vs. Summative Validation

The regulatory pathway distinguishes between two critical phases of evaluation:

- *Formative Evaluation:* Conducted iteratively during development. These studies are exploratory, allowing designers to observe how representative users interact with prototypes and to refine the interface before the design is “frozen” [6,7].
- *Summative Human Factors Validation:* Conducted at the final stage of development. This is a rigorous, objective test to demonstrate that the final finished device can be used safely and effectively by the intended population [6,7,16].

### 5.4. Regulatory Alignment with Operational Validity

For POC molecular diagnostics, successful regulatory submission (e.g., 510(k) or De Novo pathways) now increasingly depends on human factors evidence [6,16,17]. By mandating that validation include representative users and realistic environments, these frameworks operationalize the concept of operational validity. In this sense, usability engineering functions as a performance-preserving mechanism, ensuring that the analytical validity established in the lab is maintained in the hands of the end-user.

## 6. Summative Usability Testing as the Operational-Validity Benchmark (Revised)

Summative usability testing represents the definitive stage where the integrated user–device–workflow interaction is evaluated under conditions that rigorously approximate real-world use. For decentralized molecular point-of-care (POC) diagnostics, summative testing is not merely a regulatory milestone; it serves as the practical benchmark for operational validity. It empirically determines whether the intended operators can execute the full diagnostic workflow, from swabbing to reporting, correctly and safely in the noisy, high-pressure environments of actual deployment [6,7,16,17,18,19,20].

**Figure 2:**
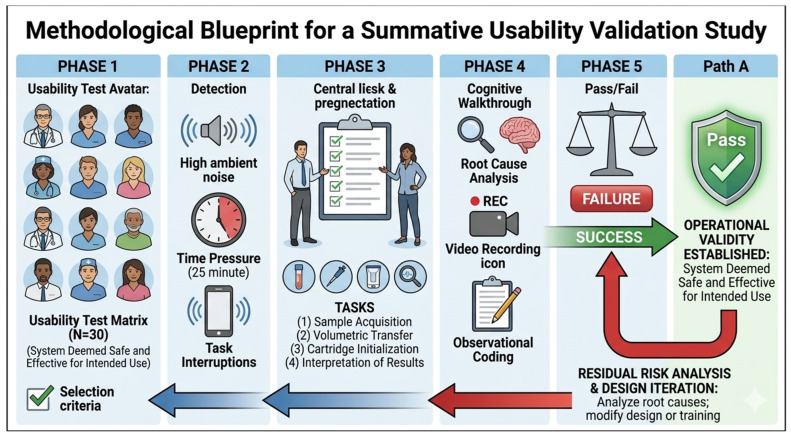
Methodological Blueprint for a Summative Usability Validation Study. This five-phase workflow evaluates medical system designs from participant enrollment through benchmarking. Phase 1 establishes the cohort via a usability matrix, while Phase 2 simulates realistic boundary conditions (noise, time pressure, interruptions). Phase 3 outlines critical device execution tasks, and Phase 4 details multi-faceted data acquisition methods. Finally, Phase 5 acts as a decision node where a “PASS” establishes operational validity (Path A), and a “FAIL” triggers residual risk analysis and design iteration.

While formative studies are exploratory and diagnostic (intended to “find and fix” design flaws), summative testing is strictly confirmatory (Figure 2). It evaluates the finalized, production-equivalent device with a statistically representative cohort of users performing predefined critical tasks. At this stage, usability is no longer a design preference; it is a validated performance specification measured against predefined success criteria [6,7,16,19,20].

From a regulatory standpoint, summative validation constitutes a core component of design validation (Table 4). It requires objective evidence that intended users, given the intended level of training, can perform critical tasks without unacceptable use errors that could lead to clinical harm or compromised results [6,16]. Methodologically, these studies translate theoretical design assumptions into quantifiable performance outcomes, effectively defining the stochastic operating envelope of the device, the boundary conditions within which its analytical sensitivity and specificity can be reliably preserved in the field [4,5,10,11,12,13,14,19,20].

## 7. Designing Simulated-Use Studies for Decentralized Settings

This section addresses the pivotal methodological challenge of operational validity: how to structure a validation study so that it meaningfully replicates the volatility of decentralized use. Regulatory guidance from the FDA mandates that validation plans provide a robust rationale for the selected environment, participant characteristics, and the “simulated-use” conditions employed [16]. Especially for Over-the-Counter (OTC) and home-use diagnostics, the FDA specifies that representative laypersons must perform tasks in environments that mirror the home setting [17]. In this context, environmental and operational realism is not a source of experimental bias; rather, it is the fundamental mechanism that reveals whether a system can preserve its validated performance under the “friction” of real-world conditions [16,17,21].

For molecular POC diagnostics, simulated-use studies must purposefully recreate the stressors most likely to destabilize the user–device interaction. These stressors are categorized into three primary domains:

- *Environmental Stressors:* Ambient noise, suboptimal lighting, and temperature/humidity fluctuations that challenge both reagent stability and user concentration [13,14].
- *Operational Stressors:* High-velocity workflows characterized by frequent interruptions, multitasking, and acute time pressure [16,19,20].
- *Cognitive/User Stressors:* Minimal training, end-of-shift fatigue, and the “automation bias” that occurs when users over-rely on instrument prompts [19,20,22].

Simulation-based validation is a mandatory regulatory mechanism because it allows investigators to observe the propensity for error in a controlled yet naturalistic setting. Unlike a standard clinical trial that might focus on diagnostic accuracy (sensitivity/specificity), a simulated-use study focuses on procedural fidelity. It exposes latent failure modes, such as a user’s tendency to skip a homogenization step when interrupted, that may never emerge in a pristine laboratory setting but would result in a false-negative result in a busy pharmacy or urgent care clinic [10,11,12,13,14,16,17,18,19,20,21,22]. By intentionally “stressing” the system, researchers define the limits of its operational validity, ensuring the device is resilient enough for the settings in which it is intended to function (Table 5).

## 8. Linking Usability Factors to Diagnostic and Clinical Outcomes

**Figure 3:**
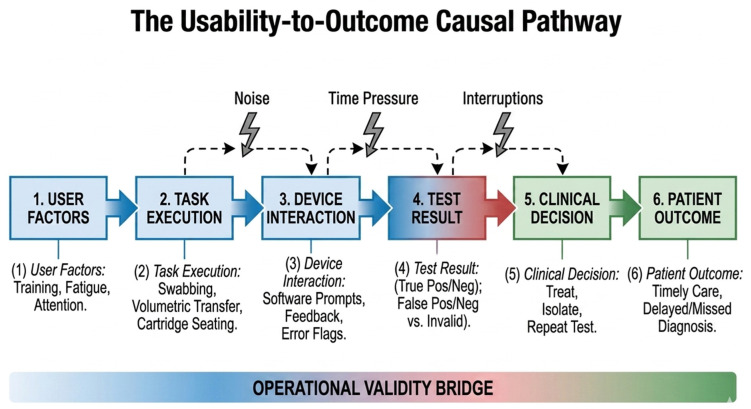
The Usability-to-Outcome Causal Pathway. This model illustrates how human factors and environmental stressors (noise, time pressure, interruptions) sequentially propagate through task execution and device interaction to affect test accuracy. The underlying “Operational Validity Bridge” signifies that clinical utility depends on the entire user-device-environment chain remaining intact.

Human factors become clinically significant only when they alter the trajectory of the diagnostic pathway. In decentralized molecular diagnostics, this pathway is not a simple binary of “test performed/result generated”; rather, it is a complex sociotechnical chain: User Factors → Task Execution → Device Interaction → Test Result → Clinical Decision → Patient Outcome (Figure 3) [3,4,14]. This dependency dictates that a test may be analytically flawless in a controlled laboratory yet fail to deliver clinical utility if usability barriers interrupt the workflow, delay reporting, or distort result interpretation [3,4,14].

The most salient evidence of this downstream clinical impact is found in user-induced sample failures. The molecular diagnostic literature consistently identifies errors in specimen collection, transport, and buffer homogenization as primary drivers of discordant results [10,23]. Suboptimal biological sampling frequently acts as a definitive cause of false-negative outcomes, often rooted in a lack of anatomical understanding or procedural haste during non-professional swab execution [11,12,23]. These pre-analytical use errors do not simply produce an isolated incorrect value; they completely derail patient management, leading to missed infections, delayed treatment, and the need for costly redundancy strategies like reflex laboratory testing [11,23].

Beyond the specimen, workflow and interface failures introduce significant operational drag (Table 6). In high-acuity environments like the Emergency Department (ED), user-dependent errors, such as cartridge misloading or process deviations, are inversely correlated with user experience and directly correlated with increased turnaround times (TAT) and prolonged ED length of stay [23]. When environmental stressors like high humidity or temperature extremes are added, even minor usability flaws in reagent handling can be amplified into catastrophic system failures [13]. These real-world observations reinforce the reality that field performance is a function of observed behavior, not just the idealized specifications in a package insert [14].

Ultimately, operational validity is the clinical safeguard of the diagnostic process. It ensures that the system is resilient enough to support timely, correct, and actionable results despite the chaos of decentralized care. By embedding usability validation into the development lifecycle, manufacturers move beyond producing a “technically valid signal” to delivering a clinically reliable tool [3,4,14,23].

## 9. Integrating Human Factors Across Design, Validation, Deployment, and Post-Market Surveillance

**Figure 4:**
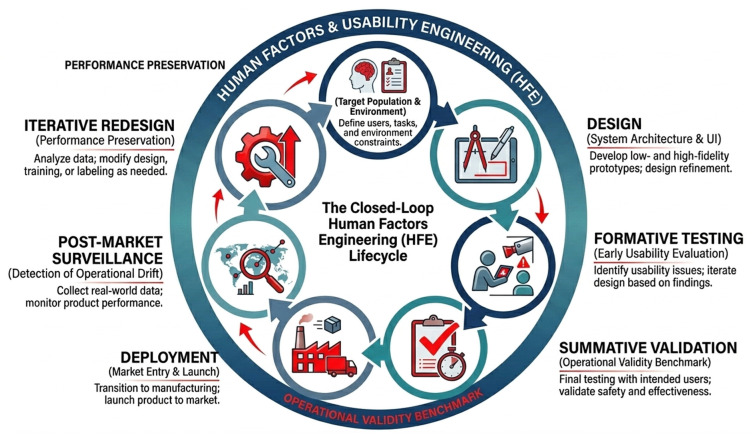
The Closed-Loop Human Factors Engineering (HFE) Lifecycle. This circular lifecycle diagram illustrates the continuous, iterative nature of Human Factors and Usability Engineering throughout the lifespan of a medical product. Unlike linear development models, this closed-loop approach ensures that real-world performance data consistently informs design improvements.

Human factors engineering (HFE) must be treated as a continuous lifecycle process rather than a discreet (Figure 4), one-time compliance activity. In decentralized molecular diagnostics, the device, the operator population, and the use environment are dynamic variables; shifts in any of these can lead to operational drift, eroding the validity established during pre-market summative testing [4,6,7,9,19,20]. A platform that demonstrates high fidelity in a supervised clinical trial may behave unpredictably in a retail pharmacy or home-testing context, where users face different cognitive loads, workflow pressures, and expectations for result escalation [4,8,14,16,17,21]. Consequently, usability is never “solved” at launch; it must be systematically monitored and maintained throughout the entire product lifecycle [6,7,9,19,20].

The HFE lifecycle begins with foundational Concept & User Research and task analysis to characterize the intended use-space, defining the operational boundary conditions the system must navigate. This is followed by Design & Risk Analysis to map critical tasks and compile the Use-Related Risk Analysis (URRA). Next, Formative Evaluation provides an iterative process where prototype interfaces, labeling, and Instructions for Use (IFU) are refined based on observed error patterns [6,7,19,20]. In molecular POC systems, this stage is critical for mitigating the risks inherent to complex tasks such as volumetric specimen transfer or multi-step cartridge initialization before they reach the field [4,5,10,11,12,13,14].

While Summative Validation serves as the benchmark for operational validity, the HFE process must extend into the post-market phase (Table 7). Once deployed, a device’s performance can shift as training decays, staffing turns over, or new clinical workflows evolve [4,14,19,20]. Effective lifecycle management treats post-market surveillance, including complaint trends, invalid-run frequencies, and field error reports, as active HFE inputs to detect interface degradation. Finally, under a framework of Continuous Lifecycle Risk Management, these real-world data points trigger a “closed-loop” feedback mechanism, leading to enhanced Quick Reference Guides (QRG), software UI optimizations, or hardware architecture improvements. This comprehensive approach ensures that operational validity is not merely a pre-market snapshot in time but a sustained characteristic of the diagnostic system over prolonged operational lifecycles [6,7,19,20,21,22].

## 10. Conclusions and Future Directions

Point-of-care (POC) molecular diagnostics represent a transformative shift in healthcare delivery, yet they will only fulfill their clinical promise if usability is elevated from a secondary design consideration to a core determinant of performance [4,5,6,7,9,19,20]. Across the expanding landscape of decentralized care, the fundamental question is no longer whether an assay works in principle, but whether its validated performance can survive the transition to the hands of a lay user in a non-standardized environment [4,6,7,8,9,14,16,17,18].

This review’s central contribution is the formalization of operational validity as the preservation of intended diagnostic performance under real-world use conditions. By framing usability as a mechanism of performance preservation, we bridge the gap between analytical excellence in controlled settings and clinical reliability in the field [1,2,3,4,10,11,12,13,14]. Under this framework, summative usability testing and high-fidelity simulated-use studies are not peripheral regulatory hurdles; they are the primary empirical methods for demonstrating that a diagnostic system is fit for purpose [6,7,16,17,18,19,20,21,22].

### 10.1. Practical Implications for Stakeholders

The implementation of a formalized operational validity framework carries distinct, practical imperatives for the diagnostic ecosystem:

- For Manufacturers: HFE can no longer be approached as a late-stage, check-the-box regulatory hurdle. Product teams must integrate iterative formative testing early in the design phase to build “error-tolerant” architectures, thereby reducing expensive late-stage summative validation failures, lowering cartridge wastage rates, and ensuring market readiness under the stringent May 2026 FDA marketing submission guidelines.
- For Regulators: This framework supports a shift toward demanding objective, simulation-based behavioral data over passive compliance checklists. It provides a structured methodology to evaluate how environmental and cognitive stressors impact real-world device safety and effectiveness during 510(k), De Novo, and PMA reviews.
- For Clinicians and Healthcare Systems: Operational validity metrics provide a reliable procurement benchmark. By evaluating a platform’s user-induced invalid rates and workflow-driven turnaround time (TAT) vulnerabilities prior to deployment, clinical administrators can accurately predict actual operational drag, training decay, and nursing labor burdens in high-acuity environments.

### 10.2. SWOT Analysis of the Operational Validity Framework

To guide future strategic implementation, a comprehensive SWOT analysis reveals the balanced internal and external dynamics of applying operational validity principles to decentralized molecular testing.

The primary **strengths** of this framework lie in its ability to directly bridge laboratory analytical sensitivity with field clinical utility, minimize real-world pre-analytical use errors and cartridge waste, and establish robust, defensible compliance pathways for 2026 FDA submissions. However, these advantages are coupled with notable internal **weaknesses**, including increased initial R&D costs, prolonged development timelines, high complexity in designing high-fidelity simulated-use environments, and an inherent difficulty in gathering standardized, peer-reviewed field error data.

Looking outward, substantial **opportunities** exist to drive the engineering of fully automated, layperson-tolerant diagnostics, foster the creation of cross-industry standardized usability metrics, and accelerate safe, decentralized market expansion into over-the-counter home care. Conversely, the framework must navigate significant external **threats**, such as fragmented international regulatory environments that slow global adoption, rapid market scaling that outpaces user interface (UI) design controls, and the proliferation of “unsubstantiated algorithmic claims” or “hallucinatory health claims” via unvalidated AI diagnostics.

### 10.3. Future Directions: The Era of “Invisible” Complexity

As molecular diagnostics move deeper into the home and community, the next generation of POC systems will likely become more operationally demanding.

- *At-Home Expansion*: The proliferation of over-the-counter (OTC) molecular tests necessitates a new standard for “error-tolerant” design, where the interface must compensate for a total lack of professional supervision [15,17].
- *AI and Connected Health*: While AI-assisted interfaces may simplify result interpretation, they introduce novel human-device interaction risks—such as automation bias and the potential for unsubstantiated algorithmic claims or hallucinatory health claims. This necessitates the implementation of transparent, Explainable AI (XAI) frameworks and standardized Probiotic-style Model Cards during HFE evaluation to ensure users remain critical, objective evaluators of test data [5,6,8,9].
- *Syndromic-Multiplexing*: The transition from single-target assays to expansive syndromic multiplexing panels increases the cognitive burden of result interpretation. Context-specific operational validity will increasingly depend on the system’s ability to present complex multi-target data clearly without triggering user confusion, automation bias, or clinical misaction [4,5,10,11,12,13,14].

### 10.4. A Call for Standardized Metrics

A major priority for the field is the harmonization of operational validity metrics. While current regulatory frameworks establish the *process* of usability engineering, there is a lack of standardized reporting for task success rates, critical-task failure frequencies, and “user-induced” invalid-run rates in peer-reviewed literature [19,20,21,22]. Establishing these benchmarks will allow for more transparent comparisons between platforms and a clearer understanding of how usability affects population-level health outcomes.

Ultimately, the goal is to ensure that decentralized molecular diagnostics are not only analytically sound but operationally resilient. By integrating Human Factors Engineering across the full device lifecycle, the diagnostic industry can ensure that scientific innovation translates into consistent, reliable, and actionable clinical results, regardless of where the test is performed or who is performing it [1,2,3,4,5,6,7,8,9,10,11,12,13,14,15,16,17,18,19,20,21,22].

## Figures and Tables

**Table 1:** Ergonomic and Cognitive Demands in Molecular POC Testing.

Workflow Step	Typical User Task	Cognitive/Technical Load	Foreseeable Use Error/Failure Mode	Potential Operational Consequence
Specimen Collection	Obtain swab, saliva, or blood sample	High procedural fidelity; technique-dependent	Inadequate swabbing pressure; incorrect anatomical sampling site	False-negative results due to low target biological load
Sample Preparation & Transfer	Mix specimen with buffer; transfer to cartridge	High manual dexterity; volumetric precision	Volume inaccuracies; introducing air bubbles during fluid transfer	Reduced analytical sensitivity; instrument fluidic fluid handling failure
Cartridge Handling	Inspect, orient, and secure cartridge	Spatial awareness; interpreting mechanical/actile feedback	Incomplete physical closure; contamination of sensitive fluidic ports	Instrument error codes; invalid operational runs; biohazard exposure
Instrument Interaction	Authenticate users; select test panel; monitor status	Attention to software prompts; menu interface navigation	Attentional blink; misinterpretation of user interface (UI) icons	Delayed execution; initiation of incorrect assay protocol parameters
Result Interpretation	Read qualitative output; act on invalid/error flags	Synthesis of qualitative/quantitative visual data	Confirmation bias; automation bias regarding invalid warning flags	Misdiagnosis; inappropriate clinical management decisions

This table illustrates how technical complexity is distributed across the testing sequence, emphasizing that human-system interactions are as critical to the final field result as the assay’s internal biochemistry [5,10,11].

**Table 2:** Determinants of Operational Validity: Validation Requirements.

Determinant	Impact on Reliability	Validation & Measurement Implication
Operator Variability	Execution variance; increased risk of handling errors and “workarounds”	Recruitment of representative users (non-professionals) in summative studies; use of “naturalistic” observation to identify latent errors.
Environmental Variability	Disrupted timing; compromised reagent stability; reduced interface visibility	Robustness testing under boundary conditions (e.g., high noise, low light, temperature extremes); simulated-use testing in actual field sites.
Workflow Deviations	Invalid runs; incomplete processes; increased repeat testing burden	Critical-task analysis (CTA); inclusion of intentional interruptions and time-pressure scenarios during validation trials.
Pre-analytical Variation	False negatives; reduced sensitivity; poor reproducibility	Usability validation of sampling kits; assessment of specimen-adequacy controls; quantification of “swab-to-cartridge” transfer errors.
Interpretation Burden	Misinterpretation of results; “automation bias”; delayed clinical action	Comprehension testing of the “Instructions for Use” (IFU); accuracy assessment of result-reporting tasks under cognitive load.

This table focuses on the methodological requirements for establishing operational validity, moving beyond simple error identification to formal validation strategies [4,10,11,12,13,14,15].

**Table 3:** Comparative Framework: FDA HFE Guidance vs. IEC 62366-1 [6,7,8].

Domain	FDA HFE Guidance (2016)	IEC 62366-1:2015	Operational Validity Implication
Primary Focus	Safety and effectiveness in intended use	Safety and risk management lifecycle	Moves focus from “lab specs” to “user-environment” specs.
User/Environment	Explicit characterization of user groups and settings	Formal “Use Specification” required	Essential for identifying risks in non-clinical settings (e.g., home/retail).
Task Priority	Focus on Critical Tasks (high-risk steps)	Identification of Hazard-Related Use Scenarios	Prioritizes validation on steps like sampling and interpretation.
Risk Linkage	Integrated into Design Controls	Explicitly linked to ISO 14971 Risk Management	Treats user error as a designable and mitigatable risk.
Testing Regimen	Iterative Formative + Final Summative	Required Usability Engineering process	Mandates empirical evidence over “expert opinion.”
Success Criteria	Performance of critical tasks without unacceptable error	Residual usability risks must be “acceptable”	Defines the threshold for real-world diagnostic reliability.
Output/Evidence	Human Factors Engineering Report	Usability Engineering File (UEF)	Provides the data “bridge” between the lab and the clinic.

**Table 4:** Quantitative Endpoints for Summative Usability Validation.

Metric	Context of Operational Validity	Acceptance Criterion (Risk-Based)
Critical Task Success Rate	Frequency of correct execution for high-stakes steps (e.g., sample transfer).	0 unacceptable failures; residual risks must be mitigated to “As Low As Reasonably Practicable” (ALARP).
Use Error Propagation	The extent to which an initial error (e.g., volume error) leads to a system-wide failure.	Success: System detects error (invalid flag); Failure: System produces an undetected false result.
Error Recovery Success	Ability of the user to self-correct based on UI feedback/prompts.	≥90% successful recovery for foreseeable, recoverable errors (e.g., improper cartridge seating).
Invalid-Run Rate (User-Induced)	Frequency of aborted runs due to operator handling rather than instrument failure.	Within predefined operational limits (typically ≤ 5–10% for CLIA-waived settings).
Result Interpretation Accuracy	Correct synthesis of flags, controls, and final diagnostic outputs.	≥95–99% correct interpretation across all result categories (Positive/Negative/Invalid).
Training Effectiveness	Retention of procedural knowledge after the intended training protocol.	≥90% competency achievement in first-time users post-training.

Acceptance thresholds must be justified by the manufacturer’s risk analysis. For high-acuity molecular diagnostics, the threshold for critical-task failure is typically zero, necessitating design changes if any failures are observed during the summative study [6,7,16,17,18].

**Table 5:** Modulators of Operational Validity: Experimental Stressors.

Stressor Domain	Real-World Context	Simulation Methodology	Target Usability Risk
Environmental	Busy retail pharmacy or emergency department.	Integrated background noise (70–80 dB); controlled “low-light” settings.	Missed auditory prompts; incorrect reading of visual indicators.
	Variable climate (e.g., community outreach in high heat).	Environmental chambers to test reagent/cartridge handling at the limit of IFU.	Reagent degradation; handling errors due to physical discomfort/perspiration.
Operational	Interrupted testing during concurrent patient care.	Scripted “interruptions” (e.g., a phone call) during high-risk transfer steps.	Step omission: loss of procedural sequence integrity.
	High-throughput “flu-season” surges.	Protocol-defined time limits for workflow completion.	Rushed specimen prep; skipped verification steps; volumetric errors.
Cognitive	First-time use by a layperson or minimally trained staff.	“Cold-start” testing with only the Quick Reference Guide (QRG) provided.	Inadequate label comprehension; failure to follow safety warnings.
	End-of-shift fatigue or prolonged cognitive load.	Scheduling testing sessions late in a participant’s work shift.	Attentional lapses; “auto-pilot” errors during software navigation.

The objective of simulating these stressors is not to ensure 100% success under all conditions, but to identify the point at which the diagnostic system fails to preserve its intended performance [16,17,19,20,21,22].

**Table 6:** Impact of Usability and Workflow Failures on Clinical Outcomes.

Evidence Context	Primary Usability/Workflow Failure	Quantified Diagnostic Impact	Clinical & Operational Consequence
Emergency Department POCT	User-dependent cartridge errors; improper sample handling.	6.0% erroneous results; 3.6% cartridge wastage.	Increased TAT; prolonged ED stay; avoidable nursing labor.
SARS-CoV-2 Molecular Testing	Suboptimal biological sampling; anatomical misconception.	Elevated False-Negative rates in the field.	Missed diagnosis; delayed isolation and care.
Molecular pre-analytics	Variations in collection, transport, and storage.	Reduced analytical sensitivity; false-positive/negative results.	Incorrect clinical decisions; erosion of clinician trust.
Environmental Stress Trials	Temperature/humidity exposure during reagent prep.	Reduced device reliability and assay stability.	Workflow disruption; indeterminate results requiring re-tests.
Community Paramedicine Field Study	Real-world use variability and lack of laboratory oversight.	Performance directly linked to observed user behavior.	Operational delays; diminished field-reliability of POC results.

Clinical evaluations demonstrate that usability-driven cartridge errors propagate into significant patient-flow disruptions, demonstrating that field performance is an emergent property of the user-device interaction [2,4,5,12,13,14,23].

**Table 7:** HFE Lifecycle Activities and Operational Validity Outputs [6,7,16,17,18,19,20,21].

Development Stage	Core HFE Activity	Key Output	Relevance to Operational Validity
Concept & User Research	Identify intended users, operational settings, and specific use goals	User Profiles; Use Specification; Task Map. Graphical Task Mapping Docs.	Defines the operational boundary conditions the system must navigate.
Design & Risk Analysis	Map critical tasks and foreseeable human use errors	Use-Related Risk Analysis (URRA); Preliminary Risk Controls.	Hardens the physical and software design against latent failure modes.
Formative Evaluation	Observe task performance and workflow fit	Iterative Design Refinements; Interface & IFU Optimization.	Minimizes error propagation before executing final product validation.
Summative Validation	Confirmatory usability testing under realistic clinical stressors	Human Factors Validation Report (HFVR) for Marketing Submission.	Establish the formal operational validity benchmark.
Post-Market Surveillance	Monitor field errors, invalid run rates, and runs/user feedback loop	Field Performance Data; Corrective and Preventive Action (CAPA) Corrective Action Triggers.	Detects “operational drift” and interface degradation in real-world use.
Continuous Lifecycle Risk Management	Update software UI, labeling, or hardware architectures based on field metrics	Revised Product Generation; Enhanced Quick Reference Guides (QRG).	Restores or enhances diagnostic system reliability over prolonged operational lifecycles.

## Data Availability

No new data were created or analyzed in this study. Data sharing does not apply to this article.
